# Research on the anti-interference characteristics of neural networks with different scales

**DOI:** 10.1038/s41598-025-19187-0

**Published:** 2025-10-08

**Authors:** Jie Lin, Mai Lu

**Affiliations:** Key Laboratory of Opto-Electronic Technology and Intelligent Control of Ministry of Education, Lanzhou Jiaotong University, Lanzhou, 730070 Gansu China

**Keywords:** Neural network, Anti-disturbance characteristics, Hodgkin—Huxley model, Chemical synaptic model, NW small world network structure, Biophysical models, Network models

## Abstract

The anti-interference characteristics of the neural network have a key impact on its information processing ability in complex environments. Most of the existing research focuses on small-scale networks and simplified models, and there is still a lack of systematic discussion on the influence mechanism of large-scale network expansion and topological complexity. In this study, a large-scale neural network model with different topologies is constructed to explore the influence mechanism of network size and connection complexity on the anti-disturbance characteristics. The optimal synchronization characteristics of complex NW small-world networks under noise interference are revealed, which provides a theoretical reference for the topology design and anti-interference ability improvement of artificial neural networks. Based on the Hodgkin-Huxley neuron dynamics model and Leonid chemical synapse theory, a complex Newman-Watts (NW) small-world network model containing 500 neurons is established for the first time, and the dynamic response characteristics of three topologies of simple ring network, simple NW small-world and complex NW small-world under music noise interference are compared and analyzed. The signal synchronization of the network is quantitatively evaluated by Pearson correlation, and the variation law of the anti-interference performance of different topologies is systematically revealed when the scale of the neural network is expanded from 100 to 500 neurons. The research shows that the expansion of network size and the increase of topological connection complexity can significantly enhance the anti-interference performance of neural network. Among them, the complex NW small-world network performs best in the noise interference environment, and the correlation coefficient increases significantly at the scale of 500 neurons. In this study, the network scale is extended to 500 neurons for the first time. By constructing a complex NW small-world topology, the influence of scale expansion and connection complexity improvement on the network anti-interference performance is systematically quantified, which provides reference simulation data for the simulation research of artificial neural networks.

## Introduction

The development history of brain science is essentially a process of continuous iteration of the observation accuracy and theoretical model of the nervous system. As the most complex and vital organ of human beings, the complexity of the structure and function of the brain is far greater than the ability of existing technologies to analyze any natural or artificial system. From the discovery of neuroelectric phenomena to the modeling of multi-scale network dynamics, scientists ‘exploration of brain functional mechanisms has always revolved around the spiral path of ‘observation-modeling-verification’. This process has gone through nearly two centuries of accumulation, gradually revealing the basic laws of the nervous system.

The breakthrough research in the 18th-19th centuries laid an empirical foundation for modern neuroscience. Galvani (1791) revealed the universality of bioelectricity through cross-species electrical stimulation experiments^1^, and the ion hypothesis proposed by Bernstein (1868) incorporated neural impulses into the physical and chemical interpretation framework for the first time^2^. However, limited by technical means, early studies failed to deeply analyze the microstructure of neural networks. Until Golgi’s (1873) silver staining^3^ and Cajal’s (1888) neuronal theory^4^, the morphological basis of the nervous system was established. Cajal explains the directional transmission mechanism of neural signals through the axon-dendritic polarity theory^5^, but its description of the dynamic characteristics of the network is still at the level of static anatomy, lacking systematic analysis of functional disturbance rejection characteristics.

At the beginning of the 20th century, the research focus turned to the molecular mechanism of neural signal transduction. Dale and Loewi’s discovery of the function of acetylcholine transmitter^6,7^, Sherrington’s synaptic concept^8^, and Eccles’ analysis of inhibitory synaptic ion channels^9^ jointly constructed the theoretical framework of chemical synaptic transmission. At the same time, technological innovations such as EEG^10^ and Von Békésy’s auditory mechanism research^11–14^ have promoted multi-scale cognition of brain function. Penfield’s cortical electrical stimulation map^15^ and Sperry’s split brain experiment^16^ further established the functional localization theory. However, these results mostly focus on local loop analysis, and there is still a lack of systematic discussion on the coordination mechanism of large-scale networks in interference environment.

Since the 21st century, the deep integration of multi-scale observation techniques and computational models has become a prominent feature of neuroscience. Ogawa’s fMRI technology^17,18^ and Sporns’ functional connectivity theory^19^ have realized the whole brain network analysis, while Sporns’ early research (2004) proposed the core position of small-world network in brain functional organization^20^ and further explored the association mechanism of network attributes with cognitive function and neurological diseases in the follow-up work (2013)^21^. At the level of theoretical modeling, Eccles (1964) provided a key tool for the study of neural network dynamics by simulating the Hodgkin-Huxley (HH) model^22^, describing the rhythm dynamics of the Morris-Lecar model^23^, and proposing synaptic plasticity models such as STP^24^ and STDP^25^. However, most of the existing models focus on the ideal simulation of unit mechanism or small-scale network, and there is no universal conclusion on the correlation mechanism between topology and anti-disturbance characteristics.

Neural network research has gradually focused on the anti-interference characteristics of different types and scales of network structures in complex environments, especially the potential of emerging network mechanisms and structures in improving robustness. In addition to traditional feedforward neural networks (FNNs) and recurrent neural networks (RNNs), such as Momentum Recurrent Neural Networks (MRNN) and Coevolutionary Neural Networks Considering Multiple Strategies (CNS-MS), new structures have shown the prospect of improving anti-interference ability. Momentum Recurrent Neural Networks (MRNN) improves the gradient descent algorithm of traditional RNN by introducing momentum term, which improves the convergence speed and stability, especially in dealing with long time series and noisy environment^26^. Coevolutionary Neural Networks Considering Multiple Strategies (CNS-MS) enhances the adaptability and robustness of the network through multi-strategy co-evolution. By simultaneously evolving topology, weight and learning rules, neural networks have stronger anti-interference ability in complex dynamic environments, especially suitable for reinforcement learning and evolutionary robots^27^.

In recent years, the influence of neural network topology on functional disturbance rejection characteristics has gradually become the focus of research^28^. Masland (2004)’s neuronal classification study revealed the supporting role of morphological diversity on functional modularity^29^.Lu et al. (2009) confirmed that small-world topology can improve decision stability in noisy environments^30^.Larremore et al. (2011) further quantified the impact of transmission delay on network synchronization^31^. Significant progress has also been made in the field of brain-like computing : Chen et al. (2022) enhanced the anti-interference characteristics of artificial systems through multi-objective optimization^32^, Miller et al. (2024) proved that complex network characteristics can improve the anti-interference ability of graph neural networks^33^, and Boccato et al. (2024) realized the stable transmission of spiking neural networks under electromagnetic interference through topology optimization^34^. It is worth noting that in the study of small-scale neuron system dynamics, the Man Menghua team revealed the regulation mechanism of chemical synaptic model parameter perturbation on action potential coding^35^, and verified the robustness of symbolic dynamics coding to electromagnetic interference at the hardware neuron level^36^. However, there are still some limitations in the existing research : most of the work is limited to theoretical models or small-scale network simulations, and lacks systematic verification of large-scale network scalability^37,38^. Simplified neuron models (such as the integral firing model)^39^ are difficult to truly reflect the electrophysiological characteristics of biological neurons and their regulation of network dynamics^40,41^; the coupling interference mechanism of noise has not been fully quantified.

Based on this, this study takes the Hodgkin-Huxley (HH) model^42–45^ as the core, and combines the nonlinear dynamics of Leonid chemical synapses^46^ to construct a simple ring network and a NW small-world network with a scale of 10-500 neurons^47–49^. Systematically explored the influence of topology and scale on the anti-disturbance characteristics. The innovations of this study are as follows: (1) Combining the HH model with the NW small-world topology, the dynamic response of large-scale networks under noise (sinusoidal, musical noise) interference is discussed. (2) Through the simulation study of neural networks with different complexity and various scales, the influence mechanism of topology complexity and network scale on the anti-interference ability of neural networks is analyzed. (3) Pearson correlation is used to quantify the anti-interference ability of neural network. The research results supplement the analysis of anti-disturbance characteristics of large-scale biological neural networks to a certain extent, and provide a theoretical reference for the topology optimization of artificial neural networks in complex environments.

## Materials and methods

### Hodgkin-Huxley model

The Hodgkin-Huxley (HH) model^42^ was proposed by British physicists Alan Hodgkin and Andrew Huxley in 1952. The model describes the electrical activity of neuronal cell membrane, especially the generation and propagation mechanism of action potential based on the experimental data of the process of action potential generation and conduction of squid axon cell membrane^43^. For the first time, the two concepts of ‘ion channel gating ‘and ‘dynamic change of conductance ‘are systematically introduced in this model. ‘Ion channel gating’, that is, the change of membrane potential not only affects the ion flux on the membrane, but also determines the size and direction of the ion current by affecting the activation and inactivation of the ion channel. The gating variables (such as m, h, and n) in the model reflect the activation and inactivation of ion channels, which provides an important theoretical framework for subsequent neurophysiological studies^44^. The ‘dynamic change of conductance’, that is, the change of ion channel conductance (such as sodium conductance and potassium conductance) in the model is no longer regarded as a constant, but the feedback relationship between conductance and membrane potential is revealed by the dynamic change of rate constant related to membrane potential. In addition, the adaptability and universality of the HH model make it not only suitable for the behavior simulation of single neurons, but also widely used in the research fields of large-scale neurons, nerve stimulation and drug response. Therefore, the model has become the core tool of computational neuroscience and provides great value for the prediction and analysis of subsequent neuronal behavior. Therefore, this paper chooses the HH model for modeling, in which the model describes the specific physical process through the following four ordinary differential equations:

1 $$\begin{array}{c}\left\{\begin{array}{c}C\frac{dV}{dt}={G}_{\text{Na}}{m}^{3}h\left({E}_{\text{Na}}-V\right)+{G}_{K}{n}^{4}\left({E}_{K}-V\right)+{G}_{L}\left({E}_{L}-V\right)+I\\ \frac{dn}{dt}={\alpha }_{n}\left(1-n\right)-{\beta }_{n}n\\ \frac{dh}{dt}={\alpha }_{h}\left(1-h\right)-{\beta }_{h}h\\ \frac{dm}{dt}={\alpha }_{m}\left(1-m\right)-{\beta }_{m}m\end{array}\right.\end{array}$$

Among them, V (mV) is the membrane potential of neurons, C (μF / cm^2^) is the membrane capacitance, I (μA / cm^2^) is the sum of the currents passing through the cell membrane, G_Na_, G_K_ and G_L_ (mS / cm^2^) are the maximum conductivity of sodium ion channel, potassium ion channel and leakage channel respectively, m is the parameter of sodium channel activation process, h is the parameter of sodium channel inactivation process, and n is the parameter of potassium channel activation process (dimensionless). The α function and β function are rate functions related to membrane potential and independent of time. According to the experiment, Hodgkin and Huxley obtained the transmembrane current data under different clamping voltages. The obtained α_m_, β_m_, α_h_, β_h_, α_n_, β_n_ curves were fitted to the curve, and the expression of each rate function was finally obtained^45^:

2 $$\begin{array}{*{20}c} {\left\{ {\begin{array}{*{20}c} {\alpha _{m} = \frac{{0.1\left( {V + 40} \right)}}{{1 - exp( - (V + 40)/10)}}} \\ {\beta _{m} = 4exp( - (V + 65)/18)} \\ {\alpha _{h} = 0.07exp( - (V + 65)/20)} \\ {\beta _{h} = \frac{1}{{exp( - (V + 35)/10) + 1}}} \\ {\alpha _{n} = \frac{{0.01\left( {V + 55} \right)}}{{1 - exp( - (V + 55)/10)}}} \\ {\beta _{n} = 0.125exp( - (V + 65)/80)} \\ \end{array} } \right.} \\ \end{array}$$

The specific parameters of the HH model are shown in Table 1:

**Table 1:** HH model specific parameter table.

Variable	Variable name	Variable value	Unit
C	Membrane capacitance of cell membrane	1	μF /cm^2^
G_Na_	The maximum conductivity of sodium ion channel	120	mS /cm^2^
G_K_	The maximum conductivity of potassium ion channel	36	mS /cm^2^
G_L_	Maximum conductivity of leakage channel	0.3	mS /cm^2^
E_Na_	The reverse potential of sodium ion	50	mV
E_K_	Reverse potential of potassium ion	− 77	mV
E_L_	The reverse potential of the leakage channel	− 54.5	mV

### Leonid chemical synaptic model

Chemical synapse is the connection point of information transmission between neurons through chemical signals. After the release of neurotransmitters in the presynaptic membrane, they cross the synaptic gap and bind to the receptors on the postsynaptic membrane, thus realizing the transmission and regulation of neural signals. In this paper, Leonid chemical synapse model is used for modeling.

The Leonid chemical synaptic model^46^ was proposed by neuroscientist Leonid Savtchenko and his team in 2007 to study the complex dynamic behavior of synaptic interactions in neurons, especially the dynamic characteristics of chemical synaptic transmission between neurons. The mathematical expression of the model is as follows:

3 $$\begin{array}{*{20}c} {I_{{{\text{syn}}}} = - C_{m} S_{2} \frac{{\partial \left( {V_{1} - V_{2} } \right)}}{{\partial t}} + G_{s} \left( {V_{p} - E_{s} + V_{1} - V_{2} } \right)} \\ \end{array}$$

Among them, I_syn_ represents postsynaptic current (μA / cm^2^), Cm represents membrane capacitance (μF / cm^2^), S_2_ represents postsynaptic membrane area ratio factor (dimensionless), G_s_ represents postsynaptic conductance (mS / cm^2^), E_s_ represents synaptic reversible potential (mV), V_p_ represents postsynaptic retention potential (mV), V_1_ and V_2_ represent presynaptic and postsynaptic neuron membrane potential (mV).

-C_m_S_2_ (∂ (V_1_ - V_2_) / ∂_t_) describes the capacitive current generated by the action potential of presynaptic neurons, and G_s_ (V_p_ - E_s_ + V_1_ - V_2_) describes the ionic current through the postsynaptic ligand-gated channel, which depends on the postsynaptic conductance G_s_ (mS / cm^2^).

### NW small world network topology

In 1998, Watts and Strogatz proposed a random network model and defined it as a WS small-world network^47^. The model is based on a ring network consisting of N nodes, each of which is connected to its adjacent K nodes. On this basis, each edge in the network is randomly redistributed by probability p, that is, there is at most one edge between any two nodes, and the nodes themselves are not allowed to form self-loops, so that the regular network is reconstructed into a random network.

**Fig. 1:**
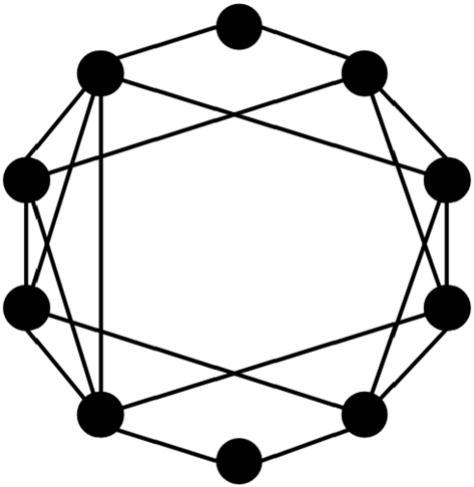
NW small world model.

However, studies have shown that the WS network is easy to generate some unrelated clustering clusters. In response to this problem, Newman and Watts improved the WS network and proposed the NW small-world network^48^, optimizing its topology and characteristics. The NW small-world network is a special topology between regular networks and random networks. Its construction idea is based on adding a small number of random connections to improve the global connectivity of the network while maintaining the regularity of the network. A key feature of the NW small-world network is that when p is small, the average path length of the network is significantly shortened while maintaining a high clustering coefficient, thus possessing small-world characteristics. This feature makes NW small-world networks widely used in biological networks, social networks, neurons and other fields. Figure 1 shows a typical NW small-world network structure^49^.

**Fig. 2:**
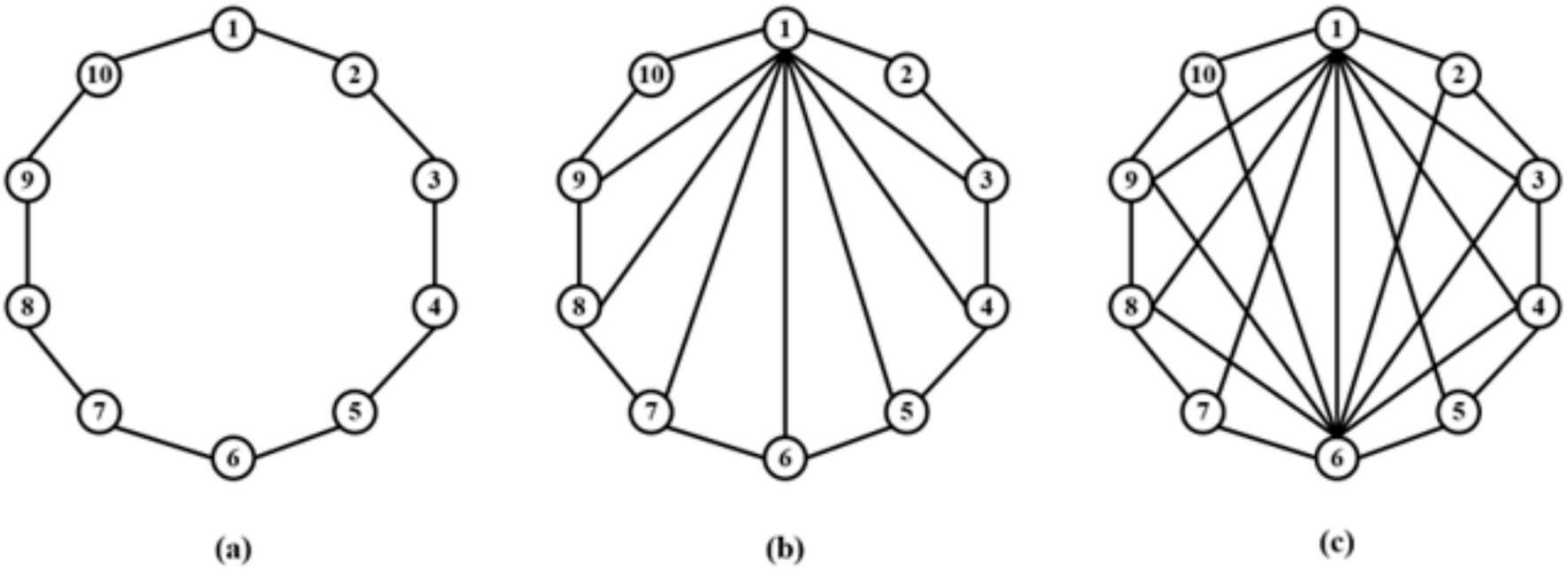
Schematic diagram of three topological structure models composed of 10 neurons.

In this study, the simple NW small-world network is based on a single ring network structure, which achieves small-world characteristics by randomly adding limited remote hop connections. On this basis, the complex NW small-world network adds a multi-connection mechanism, that is, parallel additional connection paths are introduced between several neuron pairs. This design retains the advantages of short path length and high clustering coefficient of NW network, and greatly increases the connection density. The complex NW topology shown in Fig. 2c is an extension of the simple NW topology: each neuron is connected not only to its neighboring neurons, but also to multiple distant neurons (through multiple different synapses). The experimental results show that this more complex topology can further optimize the synchronization and anti-interference performance in large-scale networks.

### Pearson correlation coefficient

In order to quantify the synchronization of neural network output signals in noisy environment, this study uses Pearson Correlation Coefficient^50,51^ as the core evaluation index of anti-interference performance. The Pearson correlation coefficient is used to quantify the linear correlation between the two signals X and Y. The calculation formula is:

4 $${r = \frac{{\sum _{{i = 1}}^{n} \left( {X_{i} - \overline{X} } \right)\left( {Y_{i} - \overline{Y} } \right)}}{{\sqrt {\sum _{{i = 1}}^{n} \left( {X_{i} - \overline{X} } \right)^{2} } \sqrt {\sum _{{i = 1}}^{n} \left( {Y_{i} - \overline{Y} } \right)^{2} } }}}$$

Among them, $\overline{X}$ and $\overline{Y}$ are the mean values, r ∈ [-1,1]. The larger the absolute value is, the stronger the correlation is, and the stronger the anti-interference performance of the network is.

### Neural network modeling

The neural network structure has many forms, and different topologies can be selected for modeling and simulation according to the research requirements. In order to explore the influence of different network structures on the dynamic behavior of neurons, this paper chooses the simple ring network structure and two NW small world network structures as the research objects. The construction of the network is based on the basic structure of neuron-synapse-neuron, in which each neuron is connected to adjacent neurons through synapses.

In the design of neural networks, topological complexity and computational cost often need to be considered together with anti-jamming performance (Manuylovich, n.d.; Ye et al., n.d.). Although increasing the number of neurons and connection density can improve the fitting ability of the network and the ability to capture signals, it will significantly increase the computational complexity and hardware resource requirements. At the same time, according to the existing research, after the network size exceeds a certain threshold, too many parameters may lead to an increase in the cumulative effect of noise, thereby reducing the anti-interference characteristics of the network. Therefore, it is necessary to strike a balance between network performance, computing resources and robustness. For example, specially designed topologies or incentive mechanisms (such as stochastic resonance neurons) can achieve better anti-noise performance with limited resources. Our results also show such similar trend. (Ye et al., n.d.).

Firstly, taking the network containing 10 neurons as an example, a simple ring network structure and a NW small world network structure are constructed by using Leonid chemical synapses. Fig. 2a shows a simple ring network model composed of 10 neurons, Fig. 2b shows a simple NW small-world network structure diagram composed of 10 neurons, and Fig. 2c shows a complex NW small-world network structure diagram composed of 10 neurons.

**Fig. 3:**
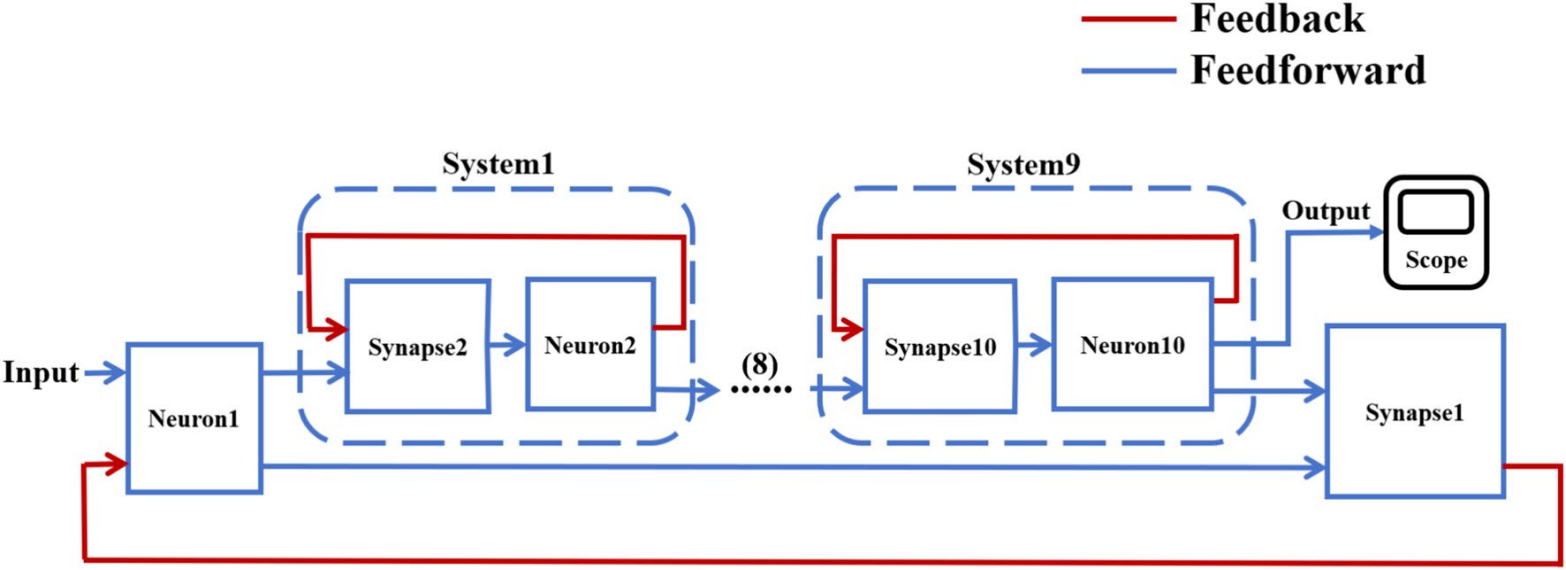
A simple ring network model composed of 10 neurons.

**Fig. 4:**
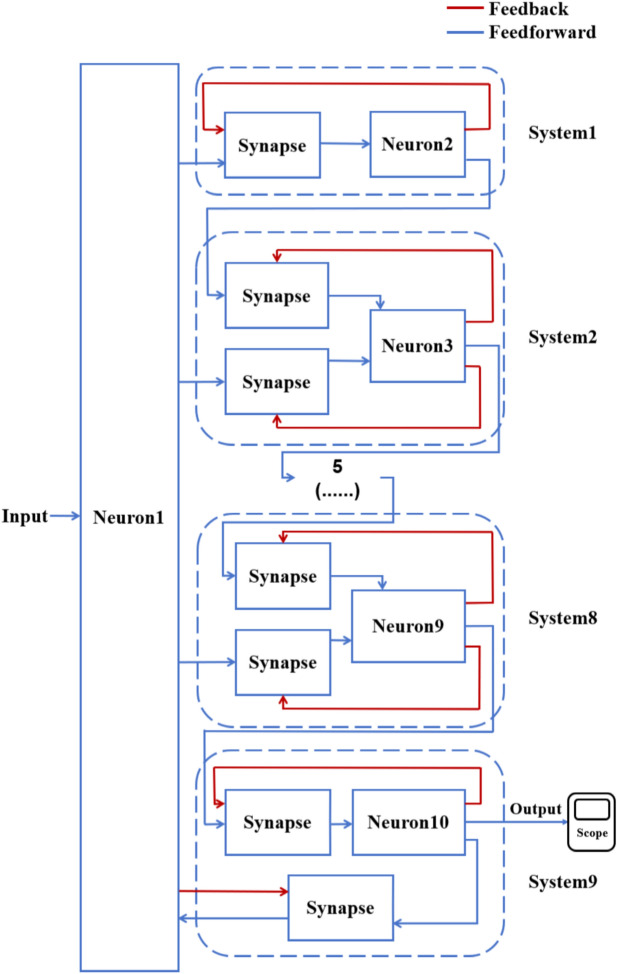
Simple NW small-world topological structure model of 10 neurons.

**Fig. 5:**
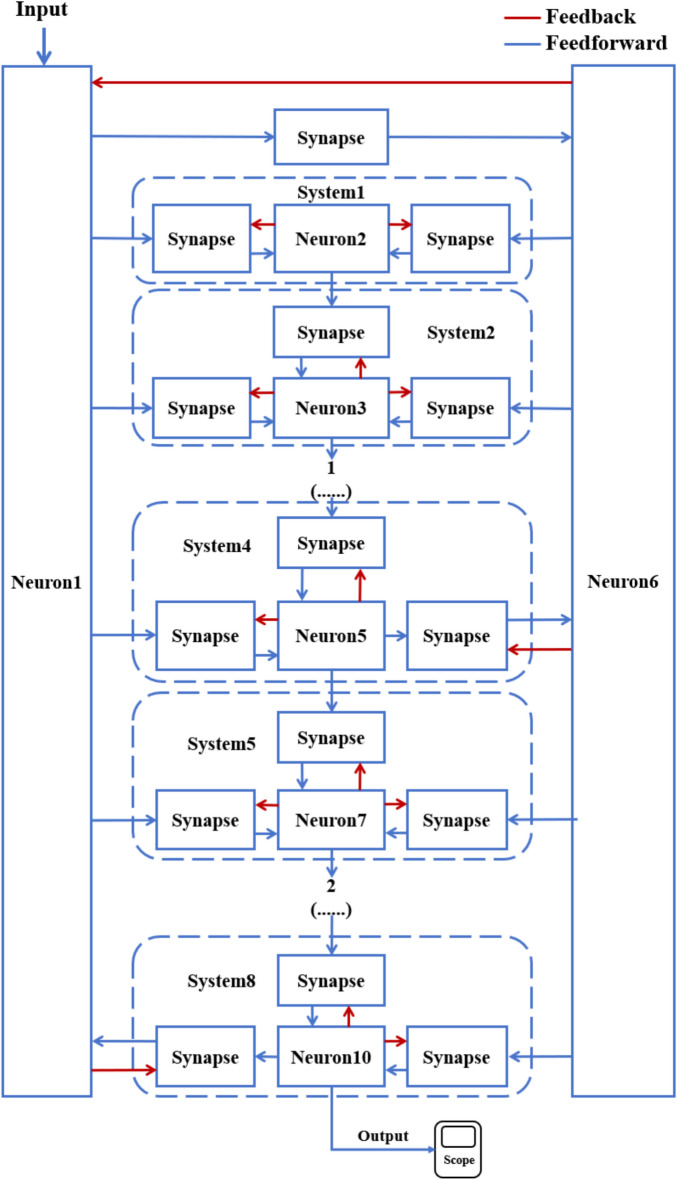
Complex NW small-world network structure model composed of 10 neurons.

According to the schematic diagram of Fig. 2, a neural network with three topological structures composed of 10 neurons is constructed. Figure 3 is a simple ring network model composed of 10 neurons. Figure 4 is a simple NW small-world network model composed of 10 neurons, and Fig. 5 is a complex NW small-world network model composed of 10 neurons. Among them, ‘Neuron1’ represents the encapsulation module of neurons, and ‘Synapse’ represents the encapsulation unit of chemical synapses.

**Fig. 6:**
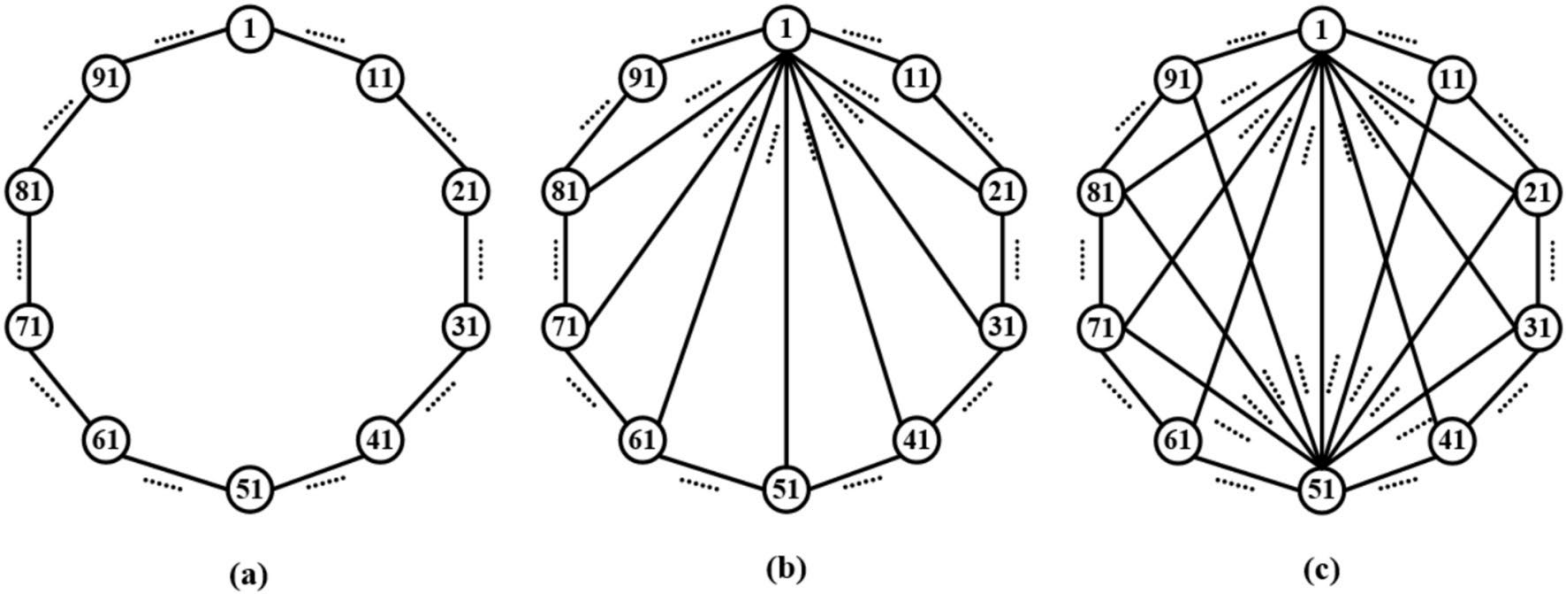
Schematic diagram of three topological structure models composed of 100 neurons.

**Fig. 7:**
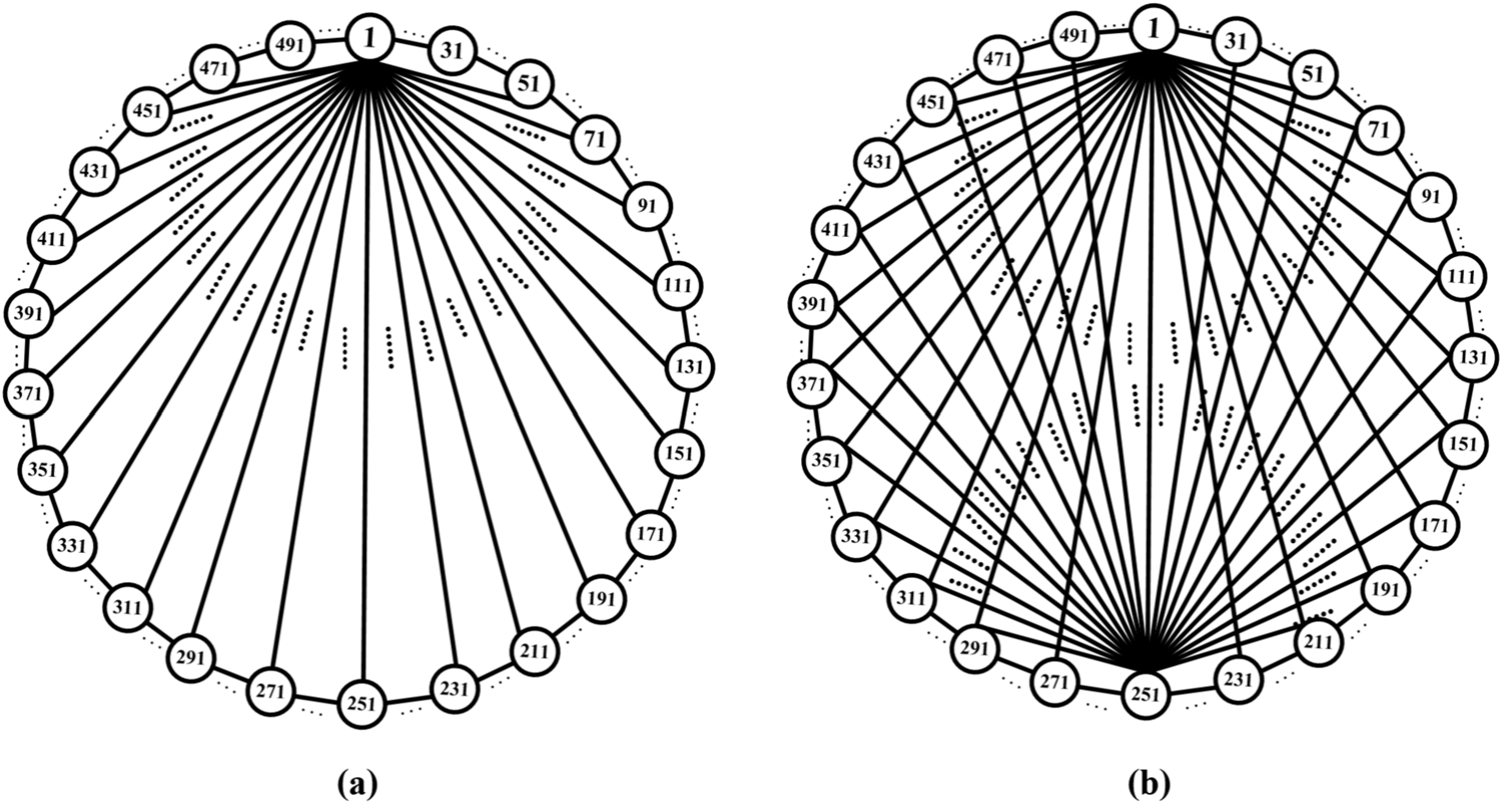
Schematic diagram of NW small-world topological structure model composed of 500 neurons.

**Fig. 8:**
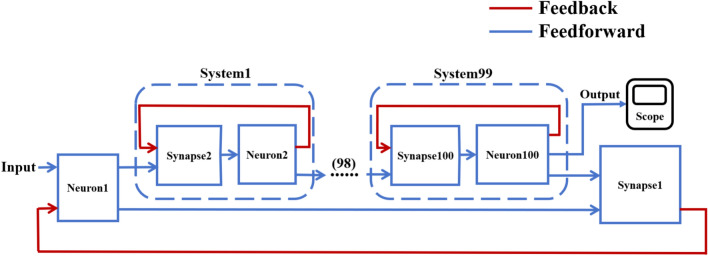
A simple ring network structure model composed of 100 neurons.

**Fig. 9:**
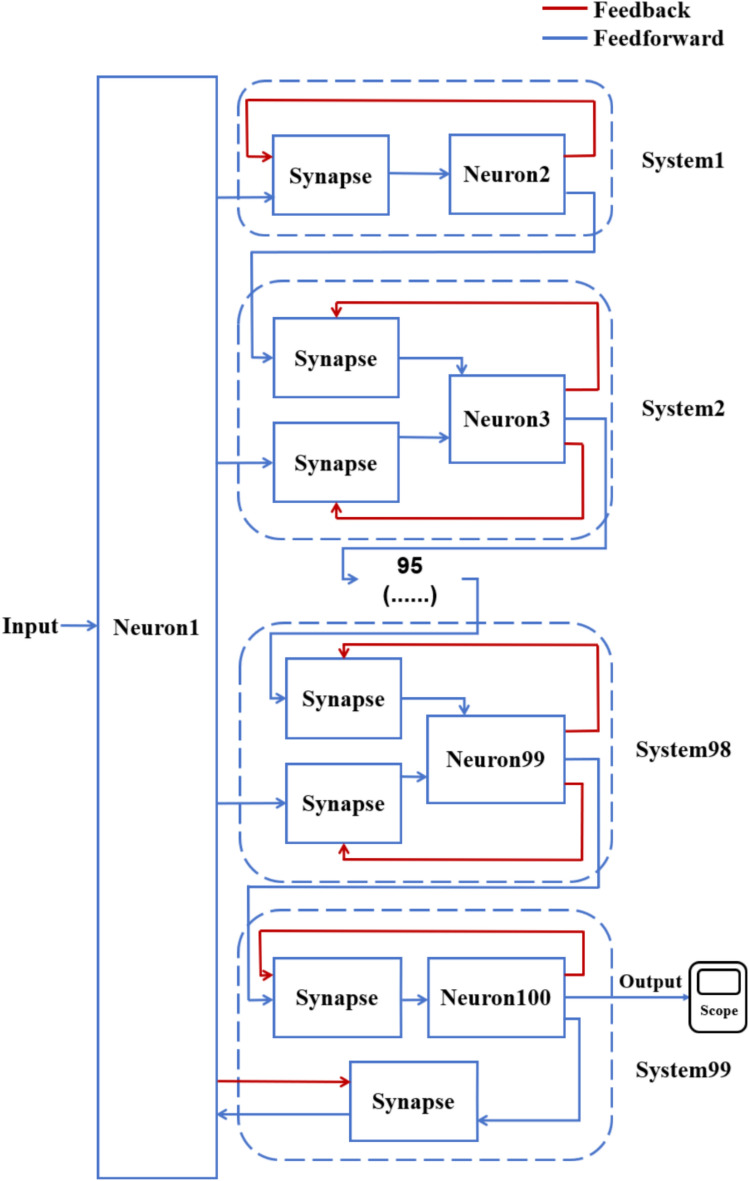
Simple NW small-world network structure model composed of 100 neurons.

**Fig. 10:**
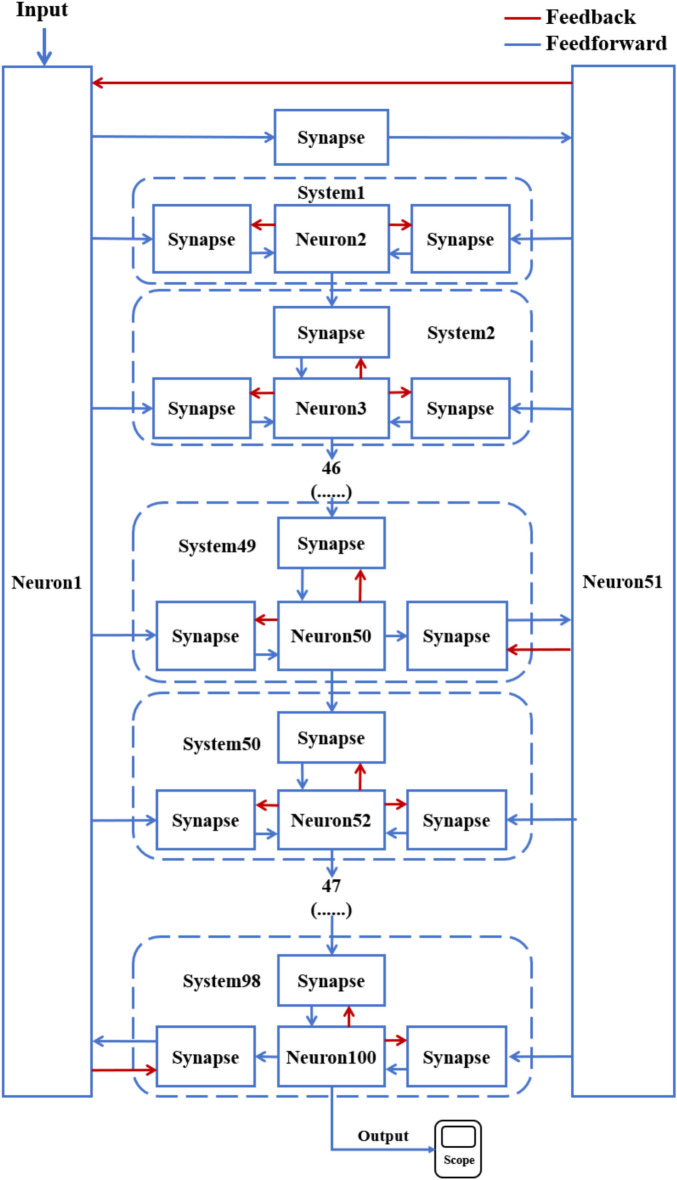
A complex NW small-world network structure model composed of 100 neurons.

**Fig. 11:**
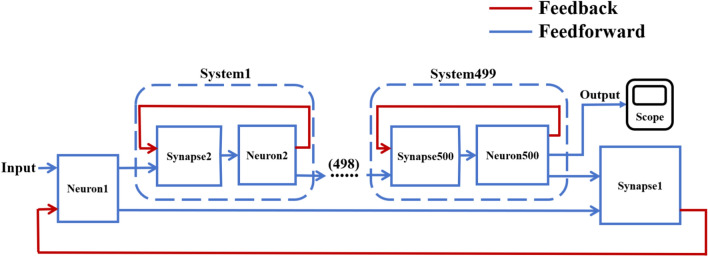
A simple ring network structure model composed of 500 neurons.

**Fig. 12:**
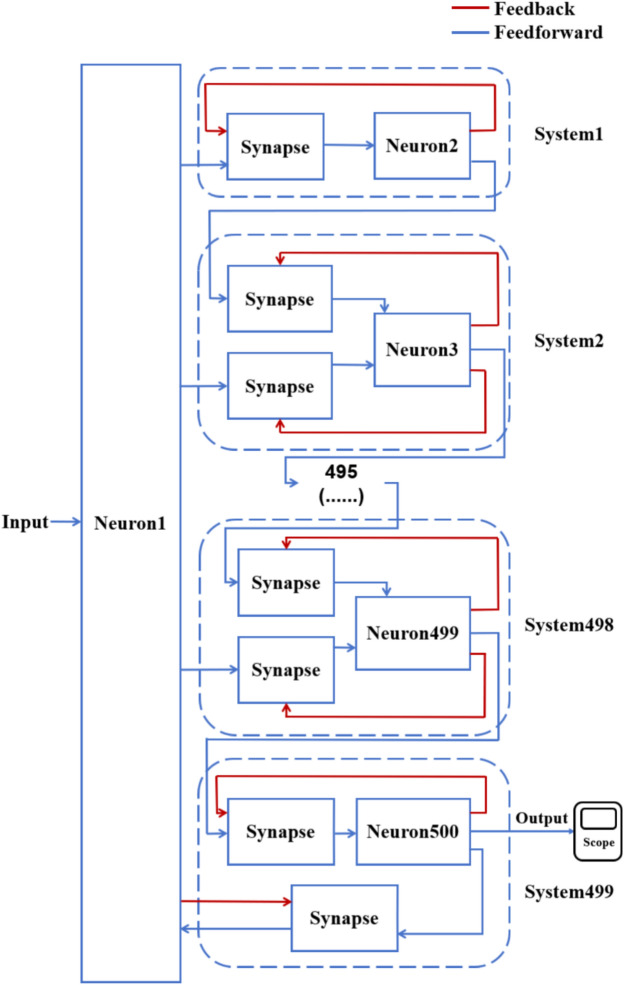
Simple NW small-world network structure model composed of 500 neurons.

**Fig. 13:**
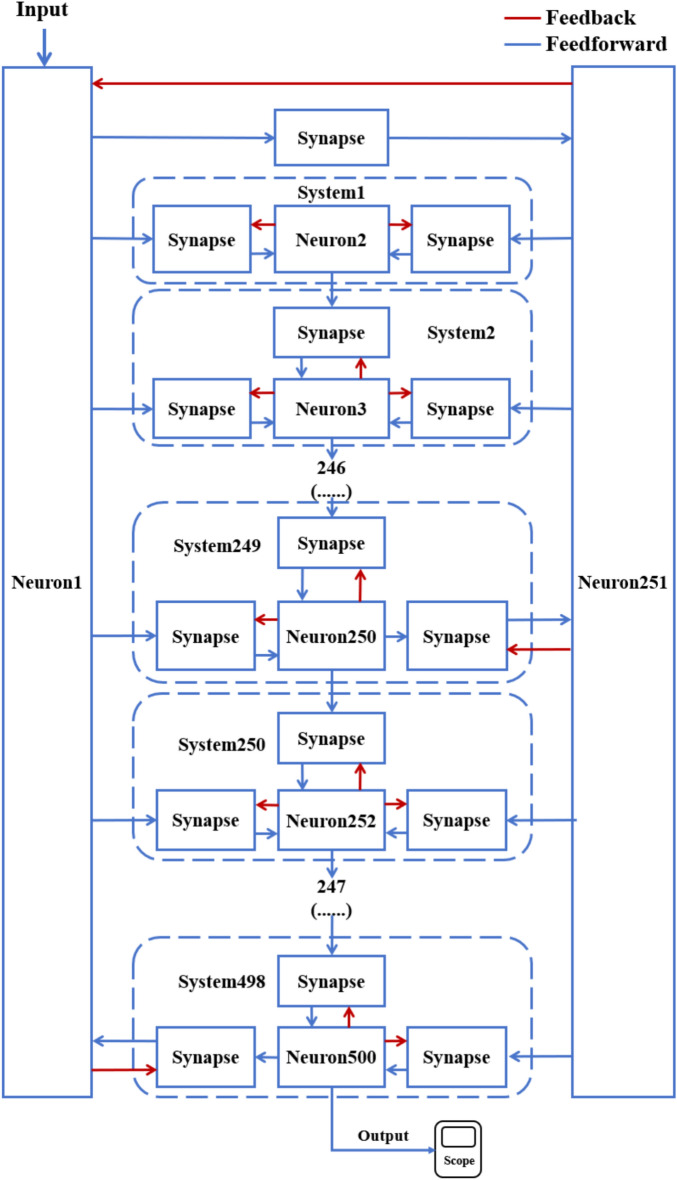
A complex NW small-world network structure model composed of 500 neurons.

On this basis, the network scale is further expanded, and a simple ring network containing 100 and 500 neurons and two NW small-world network models are constructed. Figs. 6, 7 are the schematic diagrams of three topological structure models constructed by 100 and 500 neurons, respectively. Figs. 8, 9, 10 show three topological structure models composed of 100 neurons, and Figs. 11, 12, 13 show two NW small-world topological structure models composed of 500 neurons.

## Results

### Research on the anti-interference characteristics of neural networks under different scale ring network structures

**Fig. 14:**
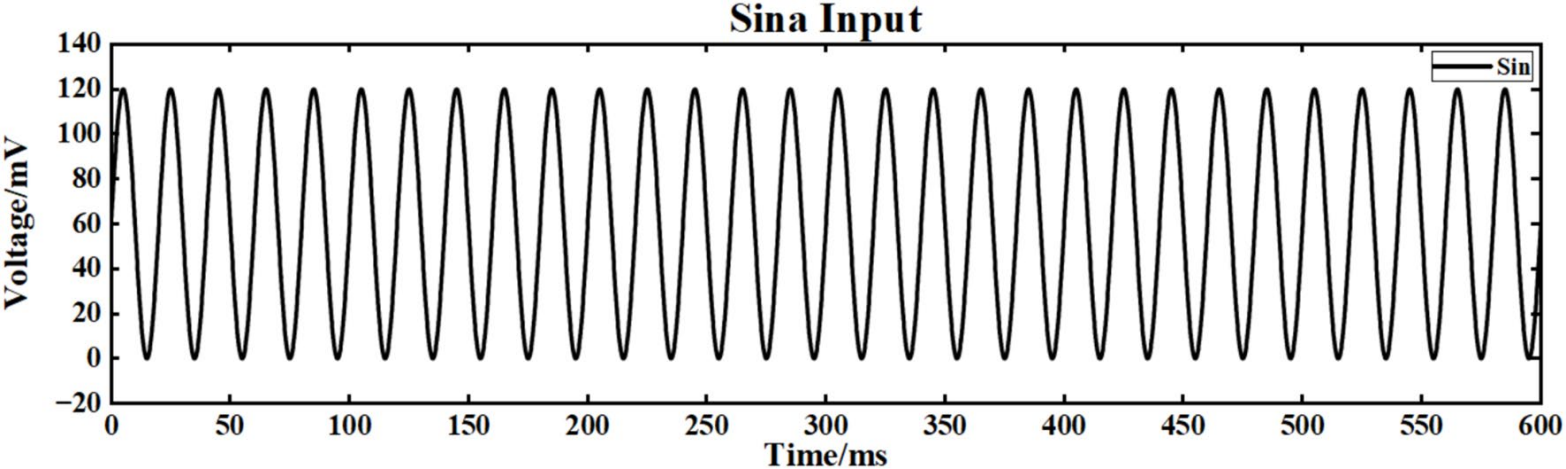
Sine input signal.

The input signal is I_ext_ = 60sin (0.1πt) + 60, and the simulation time is 600ms. The input signal waveform is shown in Fig. 14.

**Fig. 15:**
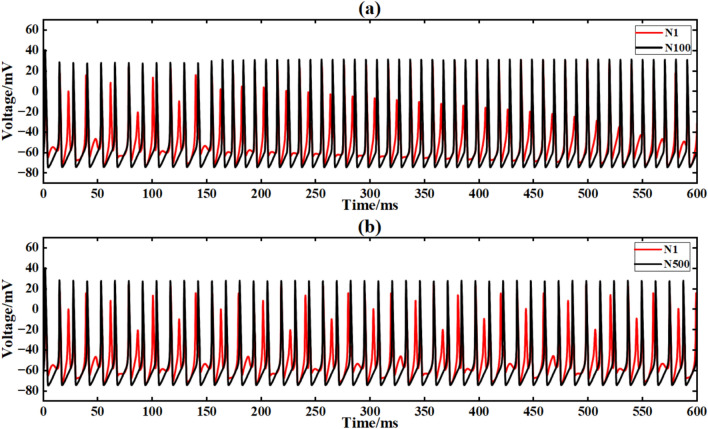
After inputting the sinusoidal signal, the output comparison diagram of neuron loop network signals of different scales is shown.

Figure 15 shows the output signal waveforms of neurons of different scales after inputting sine waves in a simple ring network structure. In the figure, (a) and (b) are the output waveforms of 100 neurons and 500 neuron ring networks, respectively, where Nx represents the output waveform of the first few neurons. By calculating the average Pearson correlation coefficient (formula 4) between the output signal of the first neuron and the output signal of the 10th, 30th, 50th, 70th and 90th neurons, the signal synchronization performance is quantitatively analyzed (Table 2).

**Table 2:** Comparison of correlation coefficients of output signals of simple neuron loop networks of different scales under input sinusoidal signals.

Number of neurons	Correlation coefficient
100 neurons	0.121
500 neurons	0.190

The average correlation coefficients of the two scale neural networks are calculated respectively, and the final results are shown in Table 2.

Under the sinusoidal signal I_ext_ = 60 sin (0.1πt) + 60 input without superimposed noise, the Pearson correlation coefficient between the output signal of the 30th neuron and the output signal of the first neuron in the 100 neuron ring network is 0.121 (Table 2), indicating that its synchronization performance is limited by the propagation delay of a single ring path. When the network size is expanded to 500 neurons, the correlation coefficient is increased to 0.190 (an increase of 57.0 %). The advantage of the large-scale ring network is that the signal can be transmitted repeatedly in a longer ring path, thereby improving the overall synchronization (Fig. 14). However, due to the inherent one-way propagation constraints of the ring network, even if the scale is expanded, the increase in the correlation coefficient is still low (< 60 %).

**Fig. 16:**
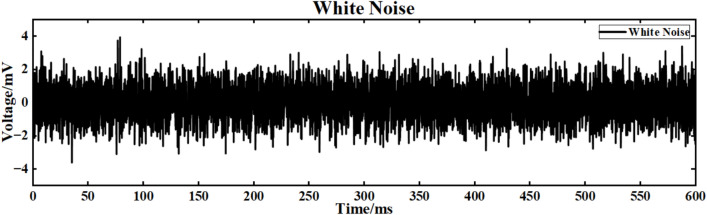
Gaussian white noise signal.

**Fig. 17:**
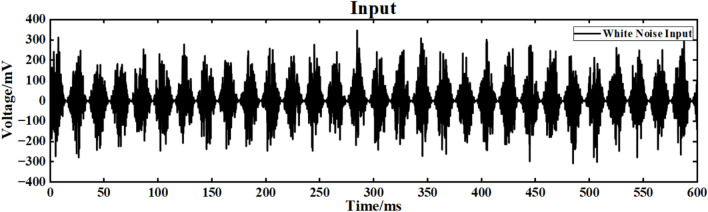
Input signal after superposition of white noise signal.

The input signal is I_ext_ = 60 sin (0.1πt) + 60 superimposed white Gaussian noise with noise power of 0.1. The simulation study of the neuron ring network structure is carried out. The simulation time is set to 600ms. The superimposed white noise signal is shown in Fig. 16, and the input waveform after the superposition of white noise is shown in Fig. 17:

Figure 20 is the output signal waveform of neurons of different sizes after adding Gaussian white noise to the input sinusoidal signal in a simple ring network structure. In the figure, (a) and (b) are the output waveforms of 100 neurons and 500 neurons, respectively, where Nx represents the output waveforms of the first few neurons.

The average correlation coefficients of the two scale neural networks are calculated respectively, and the final results are shown in Table 3.

**Table 3:** Comparison of correlation coefficients of output signals of simple neuron ring networks of different scales under input superimposed Gaussian white noise.

Number of neurons	Correlation coefficient
100 neurons	0.216
500 neurons	0.223

**Fig. 18:**
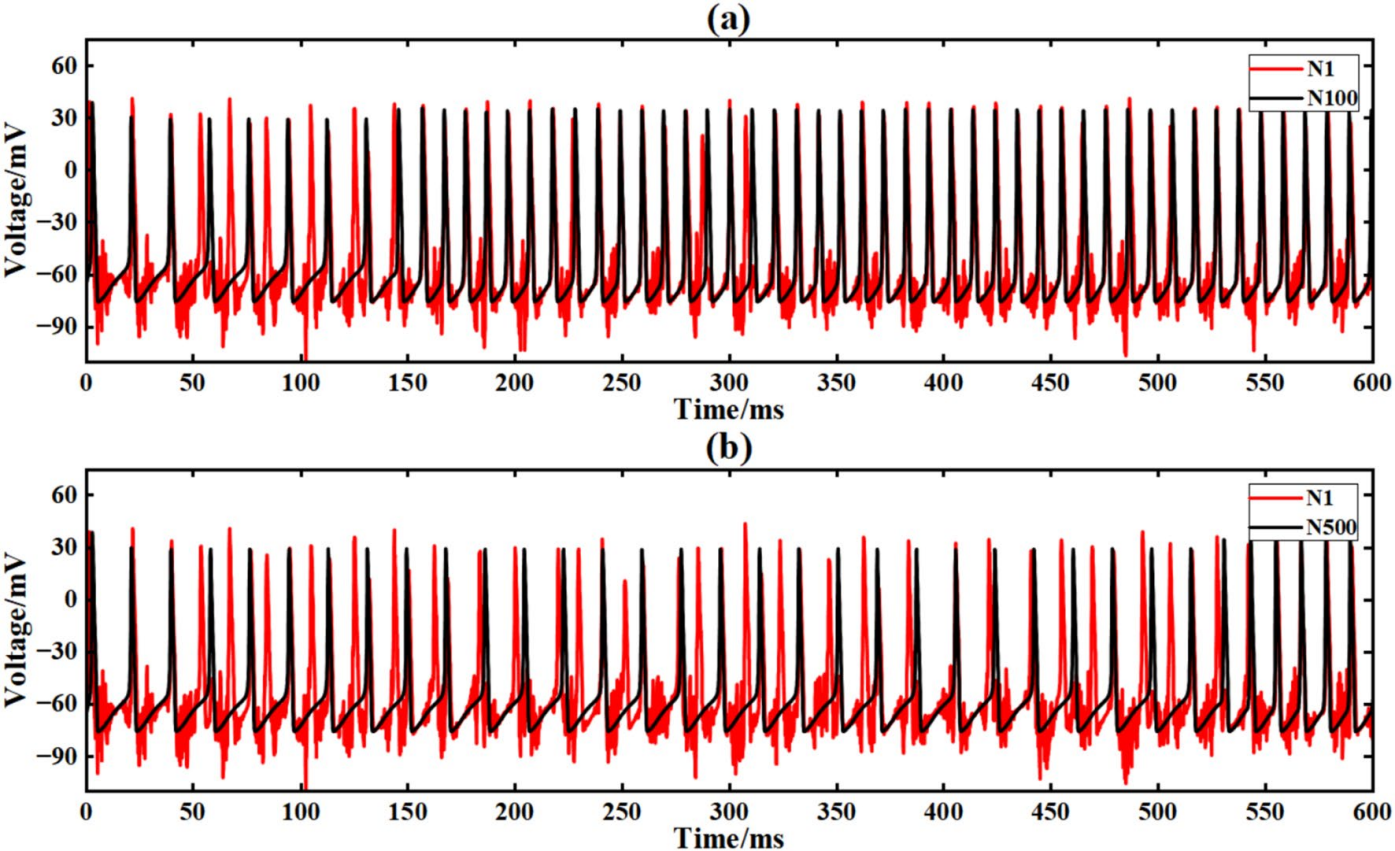
Comparison of signal output of neuron ring network with different scales after inputting superimposed Gaussian white noise.

Under the interference condition of sinusoidal signal superimposed with Gaussian white noise (Fig. 17), the correlation coefficient of 100 neuron ring networks is 0.216 (Table 3), while the correlation coefficient of 500 neuron ring networks is increased to 0.223, and the output waveform maintains obvious periodic characteristics (Fig. 18b).

**Fig. 19:**
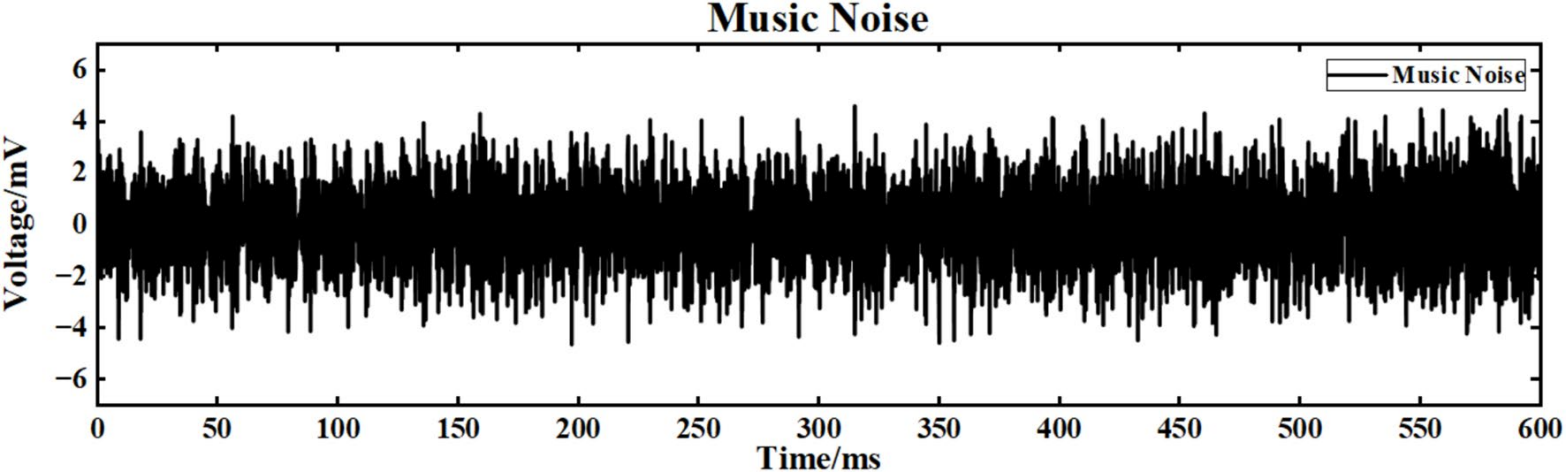
Music signal.

**Fig. 20:**
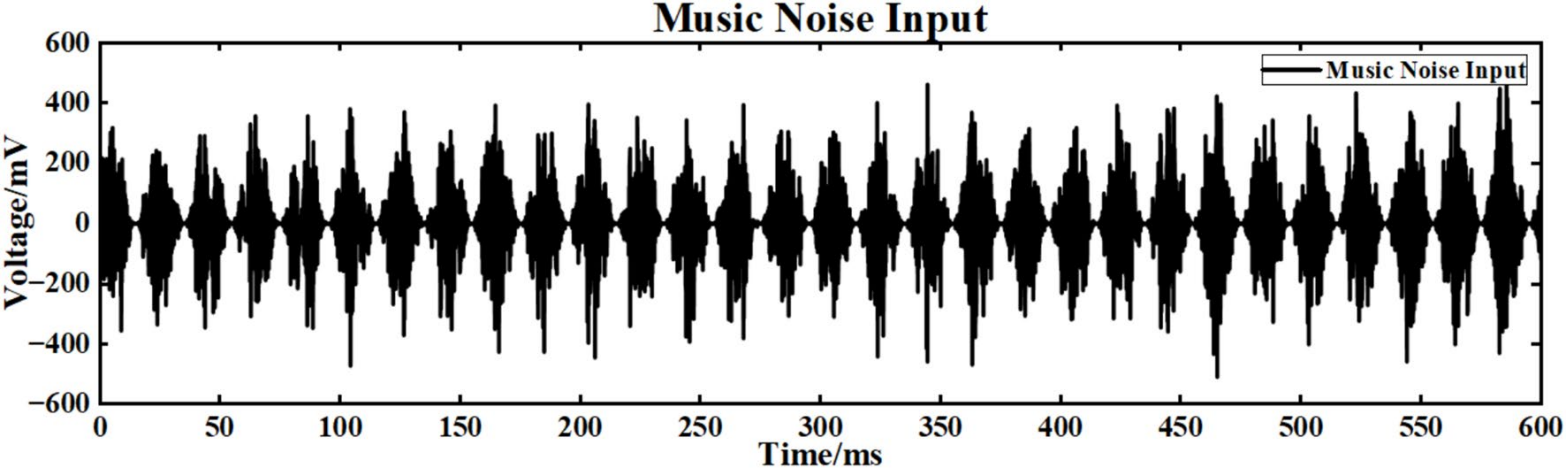
Input signal after superposition of music signal.

The input signal is I_ext_ = 60 sin (0.1πt) + 60 superimposed music noise, and the neuron ring network structure is simulated. The simulation time is set to 600ms. The music noise signal used in this study was obtained from a real music clip independently selected by us. The audio segment was imported into MATLAB and processed through sampling to generate the noise signal required for the experiments. It should be noted that this audio is not a built-in MATLAB sample but rather a music source independently extracted and preprocessed by the authors. The superimposed music noise signal is shown in Fig. 19, and the input waveform after superimposed music noise is shown in Fig. 20.

**Fig. 21:**
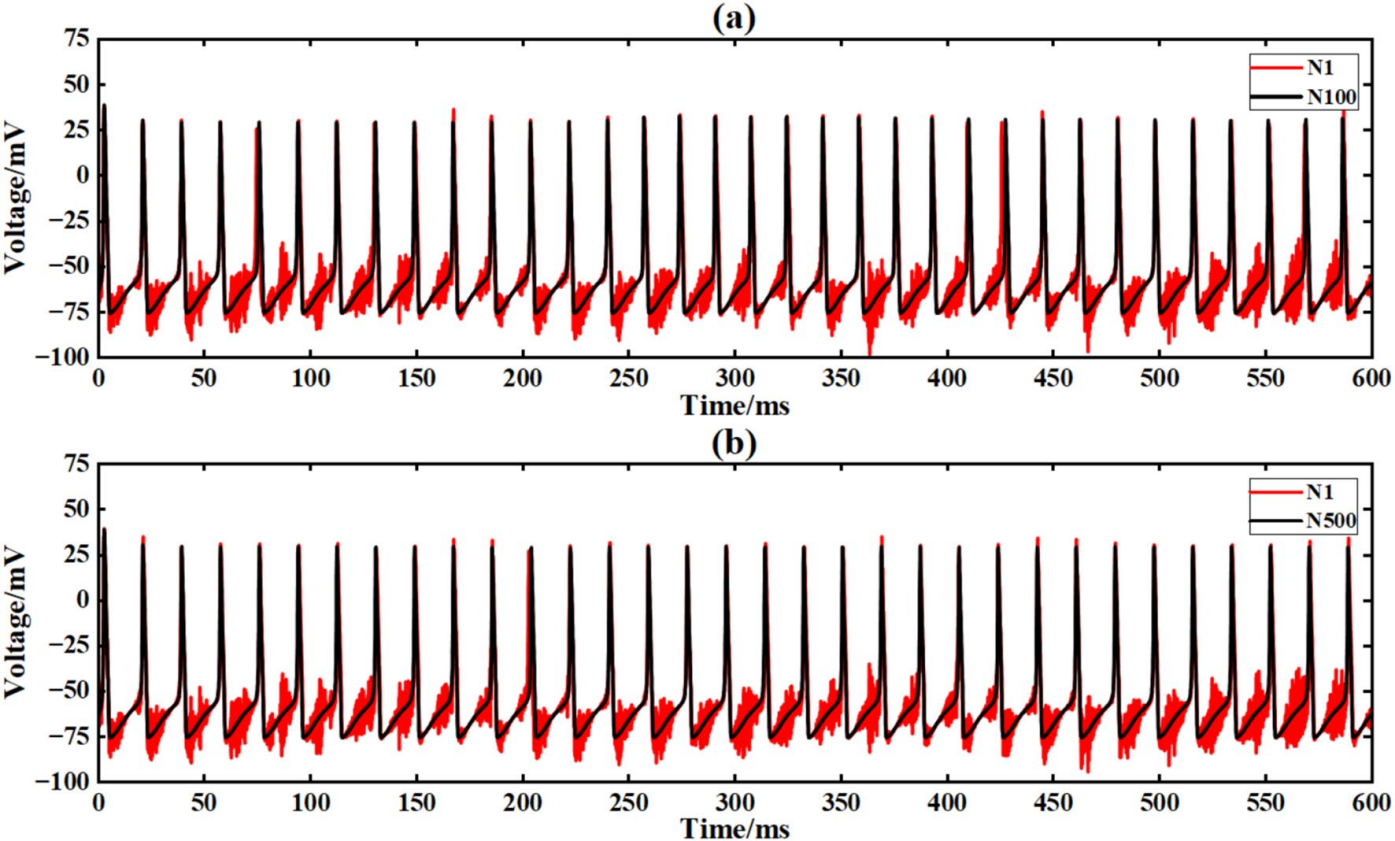
Comparison of signal output of neuron ring network with different scales after inputting superimposed music noise.

Figure 21 is the output signal waveform of different scale neurons after adding music noise to the input sinusoidal signal under the simple ring network structure. In the figure, (a) (b) is the output waveform of 100 neurons and 500 neurons respectively, where Nx represents the output waveform of the first few neurons.

The average correlation coefficients of the two scale neural networks are calculated respectively, and the final results are shown in Table 4.

**Table 4:** Comparison of correlation coefficients of output signals of simple neuron ring networks of different scales under input superimposed music noise.

Number of neurons	Correlation coefficient
100 neurons	0.240
500 neurons	0.661

Under the interference condition of sinusoidal signal superimposed with music noise (Fig. 20), the correlation coefficient of the 100 neuron ring network is 0.240 (Table 3), while the correlation coefficient of the 500 neuron network is significantly increased to 0.661 (an increase of 175 %), and the output waveform maintains clear periodic characteristics (Fig. 21b). The results show that the redundant path of large-scale ring network can weaken the interference accumulation effect by dispersing noise energy.

### Research on the anti-interference characteristics of neural networks under simple NW small-world network structures of different sizes

**Fig. 22:**
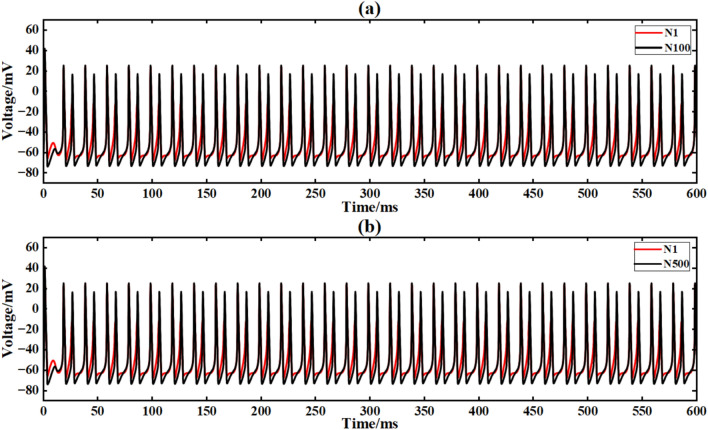
The output contrast diagram of simple NW small-world neural network signals with different scales after inputting sinusoidal signals.

The input conditions are consistent with the ring network experiment, and the network topology is constructed by a simple NW small-world network diagram (Figs. 9, 12). Figure 21 is the output signal waveform of neurons of different sizes after input sinusoidal signal under the simple NW small-world network structure. In the figure (a) (b), the output waveform of 100 neurons and 500 neuron simple NW small-world networks are respectively shown, where Nx represents the output waveform of the first few neurons. (Fig. 22).

The average Pearson correlation coefficients of several scale neural networks are obtained respectively, and the final results are shown in Table 5.

**Table 5:** Comparison of correlation coefficients of output signals of simple NW small-world neural networks with different scales under input sinusoidal signals.

Number of neurons	Correlation coefficient
100 neurons	0.812
200 neurons	0.820
300 neurons	0.937
400 neurons	0.970
500 neurons	0.989

Under sinusoidal signal input, the simple NW small-world network structure shows significantly better synchronization than the ring network structure. The calculation method of correlation coefficient is the same as that of ring network experiment, and the correlation coefficient of 100 neural network is 0.812. When it is extended to 500 neurons, the correlation coefficient further rises to 0.989 (21.8 % higher than that of 100 neurons). At this time, the signal can propagate efficiently through multiple paths and reduce phase delay.

**Fig. 23:**
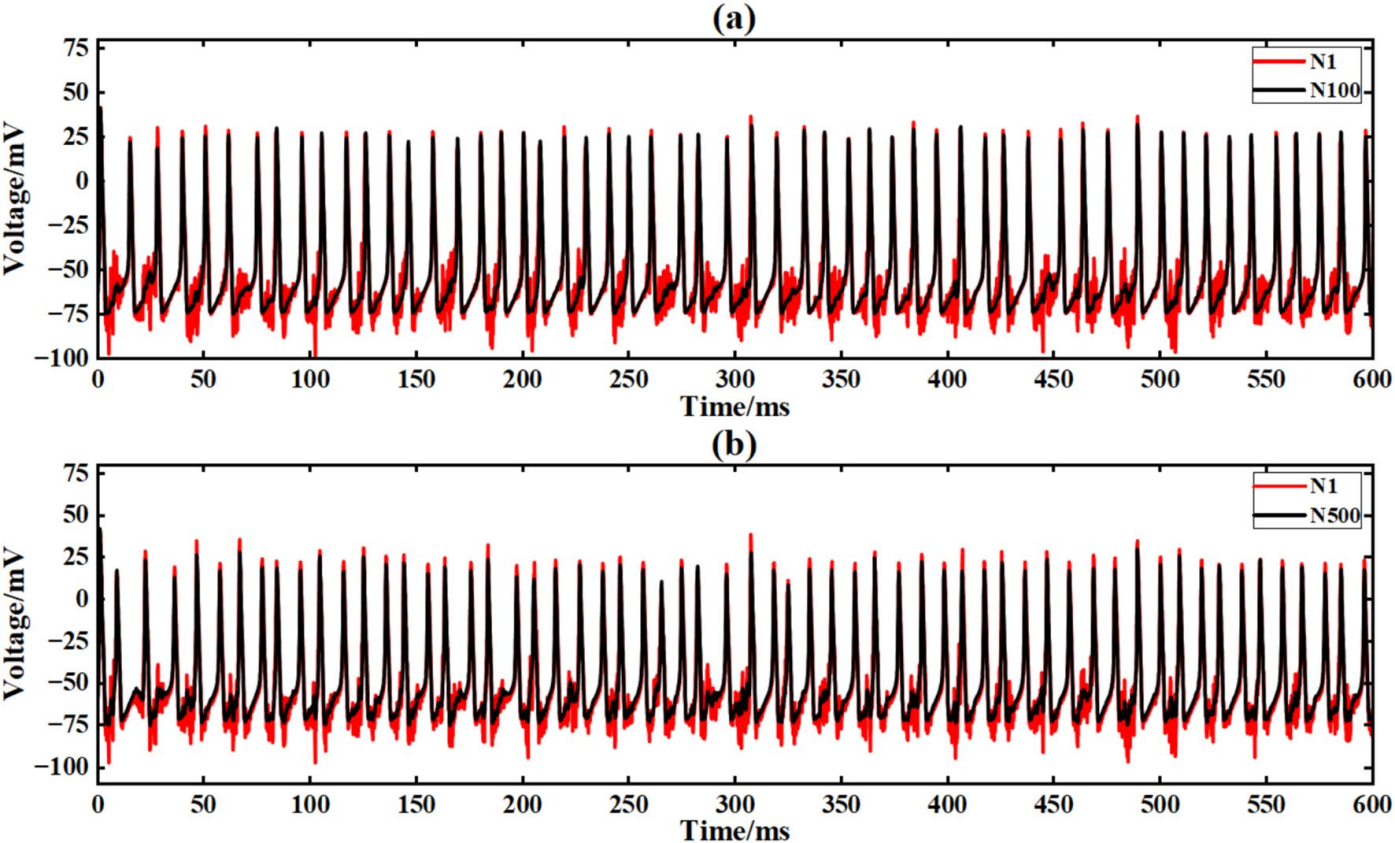
Comparison of signal output of simple NW small world network with different scales after inputting superimposed Gaussian white noise.

The input signal is I_ext_ = 60 sin (0.1πt) + 60 superimposed white noise. The neural network constructed by the simple NW small-world network is simulated. The simulation time is set to 600 ms. The superimposed Gaussian white noise signal is shown in Fig. 16. The input waveform after superimposed music noise is shown in Fig. 17. Figure 23 is the output signal waveform of neurons of different scales after the input sinusoidal signal superimposed music noise under the simple NW small-world network structure. (a) (b) in the figure are the output waveforms of 100 neurons and 500 neuron simple NW small-world networks, respectively. Where Nx denotes the output waveform of the first few neurons.

The average Pearson correlation coefficients of several scale neural networks are obtained respectively, and the final results are shown in Table 6.

**Table 6:** Comparison of output signal correlation coefficients of simple NW small-world neural networks with different scales under input superimposed Gaussian white noise.

Number of neurons	Correlation coefficient
100 neurons	0.951
200 neurons	0.955
300 neurons	0.961
400 neurons	0.965
500 neurons	0.975

When the input signal is superimposed with white noise, the NW small-world network exhibits strong noise suppression capabilities. For 100 neural networks, although noise interference still exists, the network can effectively suppress noise, and the correlation coefficient is 0.951. With the expansion of the network size, the noise suppression ability was further enhanced, and the correlation coefficient at 500 neurons increased to 0.975, an increase of 2.5%. It shows that the NW small-world network can effectively disperse and suppress the interference of white noise.

**Fig. 24:**
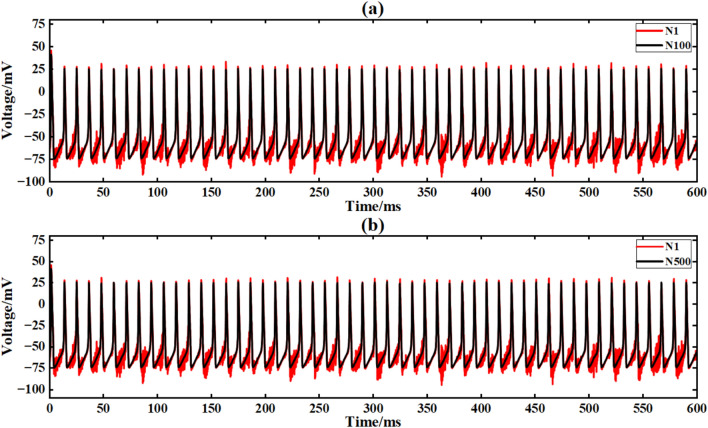
Comparison of signal output of simple NW small world network with different scales after inputting superimposed music noise.

The input signal is I_ext_ = 60sin (0.1πt) + 60 superimposed music noise. The neural network constructed by the simple NW small-world network is simulated. The simulation time is set to 600ms. The superimposed music noise signal is shown in Fig. 19, and the input waveform after the superimposed music noise is shown in Fig. 20. Figure 24 is the output signal waveform of neurons of different sizes after the input sinusoidal signal superimposed music noise under the simple NW small-world network structure. In the graph, (a)(b) are the output waveforms of 100 neurons and 500 neuron simple NW small-world networks. Where Nx represents the output waveform of the first few neurons.

The average Pearson correlation coefficients of several scale neural networks are obtained respectively, and the final results are shown in Table 7.

**Table 7:** Comparison of correlation coefficients of output signals of simple NW small-world neural networks with different scales under input superimposed music noise.

Number of neurons	Correlation coefficient
100 neurons	0.959
200 neurons	0.964
300 neurons	0.971
400 neurons	0.978
500 neurons	0.981

When inputting superimposed music noise, the NW small-world network still exhibits strong anti-interference characteristics. For 100 neurons, although the noise suppression ability is limited, the correlation coefficient is 0.959.However, with the increase of network size, the noise component is significantly reduced, and the correlation coefficient at 500 neurons increases to 0.978, an increase of 2.0 %. It shows strong low-frequency noise suppression ability, and the correlation coefficient is 420.5 % (sinusoidal signal) and 47.7 % (noise interference) higher than that of the same-scale ring network, indicating that the expansion of network scale can significantly enhance signal synchronization.

Through the simulation study of NW small-world neural networks with different scales, it can be seen that the topological advantages of NW small-world networks are particularly significant in large-scale networks, and the expansion of network scale can simultaneously improve the anti-interference ability of neural networks.

### Research on the anti-interference characteristics of neural networks under complex NW small-world network structures of different sizes

**Fig. 25:**
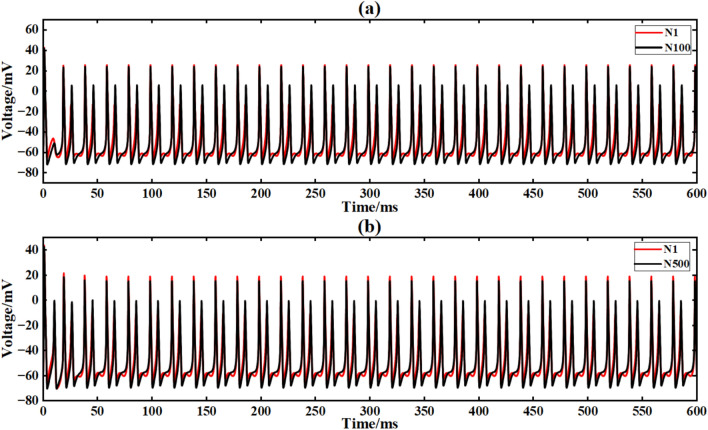
The output contrast diagram of complex NW small-world network signals of different scales after inputting sinusoidal signals is compared.

The input conditions are consistent with the ring network experiment, and the network topology is constructed by a simple NW small-world network diagram (Figsures 9and 12). Figure 25 is the output signal waveform of different scale neurons after input sinusoidal signal under the complex NW small-world network structure. In the figure (a) (b), the output waveform of 100 neurons and 500 neuron complex NW small-world networks are respectively shown, where Nx represents the output waveform of the first few neurons.

The average Pearson correlation coefficients of several scale neural networks are obtained respectively, and the final results are shown in Table 8.

**Table 8:** Comparison of correlation coefficients of output signals of two NW small-world neural networks with different scales under input sinusoidal signals.

Number of neurons	Simple NW small-world neuronal network	Complex NW small-world neuronal network
100 neurons	0.812	0.954
200 neurons	0.820	0.961
300 neurons	0.937	0.978
400 neurons	0.970	0.986
500 neurons	0.989	0.992

When the sinusoidal signal is input, the complex NW small-world network exhibits extremely strong synchronization. For 100 neurons, the correlation coefficient of the network is 0.954, indicating that the network can maintain synchronization very well. With the expansion of the scale, the synchronization of the network is further enhanced, and the correlation coefficient at 500 neurons increases to 0.992 (4.0 % higher than that of 100 neurons). Compared with the simple NW small-world network (0.989) of the same scale, the performance is further optimized by 0.3 %, which reflects the optimization effect of complex structure on the anti-interference performance of large-scale network.

**Fig. 26:**
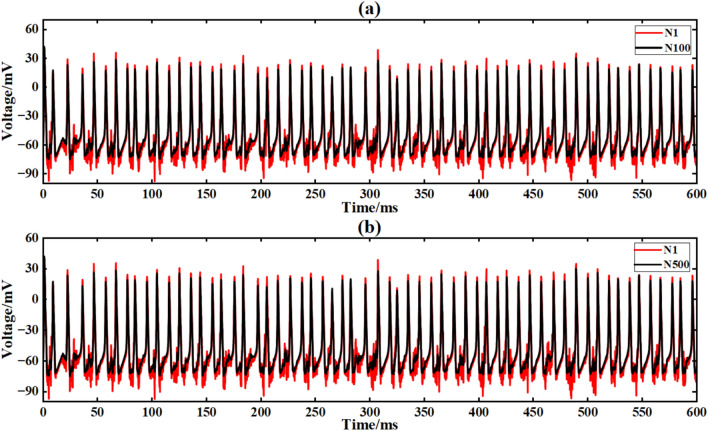
Comparison of signal output of complex NW small world network with different scales after inputting superimposed Gaussian white noise.

The input signal is Iext = 60 sin (0.1πt) + 60 superimposed Gaussian white noise. The neural network constructed by the complex NW small-world network is simulated. The simulation time is set to 600 ms. The superimposed Gaussian white noise signal is shown in Fig. 16. The input waveform after superimposed Gaussian white noise is shown in Fig. 17. Figure 26 shows the output signal waveforms of neurons of different scales after superimposed Gaussian white noise by the input sinusoidal signal under the complex NW small-world network structure. (a) (b) in the figure are the output waveforms of 100 neurons and 500 neuron complex NW small-world networks, respectively. Where Nx denotes the output waveform of the first few neurons.

The average Pearson correlation coefficients of several scale neural networks are obtained respectively, and the final results are shown in Table 9.

**Table 9:** The correlation coefficients of the output signals of two NW small-world neural networks under input superimposed Gaussian white noise are compared.

Number of neurons	Simple NW small-world neuronal network	Complex NW small-world neuronal network
100 neurons	0.951	0.962
200 neurons	0.955	0.970
300 neurons	0.961	0.973
400 neurons	0.965	0.974
500 neurons	0.973	0.976

When the input signal is superimposed with white noise, the complex NW small-world network shows better noise suppression ability. For the complex NW small-world network composed of 100 neurons, the correlation coefficient of the network is 0.962, which is 1.2 % higher than the correlation coefficient of 0.951 of the simple NW small-world network of the same size. With the increase of network size, the noise suppression ability is further improved. The correlation coefficient of the complex NW small-world network with 500 neurons increases to 0.976, an increase of 1.5 %, which is also higher than that of the simple NW small-world network of the same size. The correlation coefficient of 0.973 indicates that the complex NW small-world network can effectively cope with the interference of white noise compared with the simple NW small-world network.

**Fig. 27:**
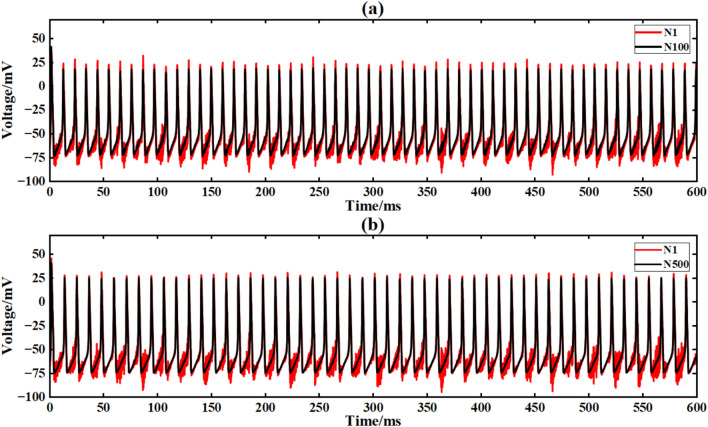
The signal output contrast diagram of complex NW small world network with different scales after adding music noise to the input is compared.

After the input signal is superimposed with music noise (Fig. 20), the complex NW small world model is simulated. The simulation time is set to 600ms. Figure 27 is the output signal waveform of neurons of different scales after the input sinusoidal signal is superimposed with music noise under the complex NW small world network structure.

The average Pearson correlation coefficients of several scale neural networks are obtained respectively, and the final results are shown in Table 10.

**Table 10:** Comparison of correlation coefficients of output signals of two NW small-world neural networks under input superimposed music noise.

Number of neurons	Simple NW small-world neuronal network	Complex NW small-world neuronal network
100 neurons	0.959	0.968
200 neurons	0.965	0.972
300 neurons	0.972	0.977
400 neurons	0.978	0.979
500 neurons	0.981	0.983

When inputting superimposed music noise, the complex NW small-world network shows stronger noise suppression ability through its more complex topology. When the number of neurons is 500, the correlation coefficient reaches 0.983, which is 0.5 % higher than that of the simple NW small-world network after adding noise. The music noise is significantly filtered out, showing a strong inhibitory effect of complex structure on noise.

The complex NW small-world network enhances the number of connections of local neurons while retaining the advantages of short paths by introducing a multiple connection mechanism. Experiments show that the topology complexity is positively correlated with the anti-interference performance. Especially in the noise environment, the structure can provide optimal anti-interference characteristics, which is suitable for application scenarios with high requirements for synchronization and anti-noise.

**Fig. 28:**
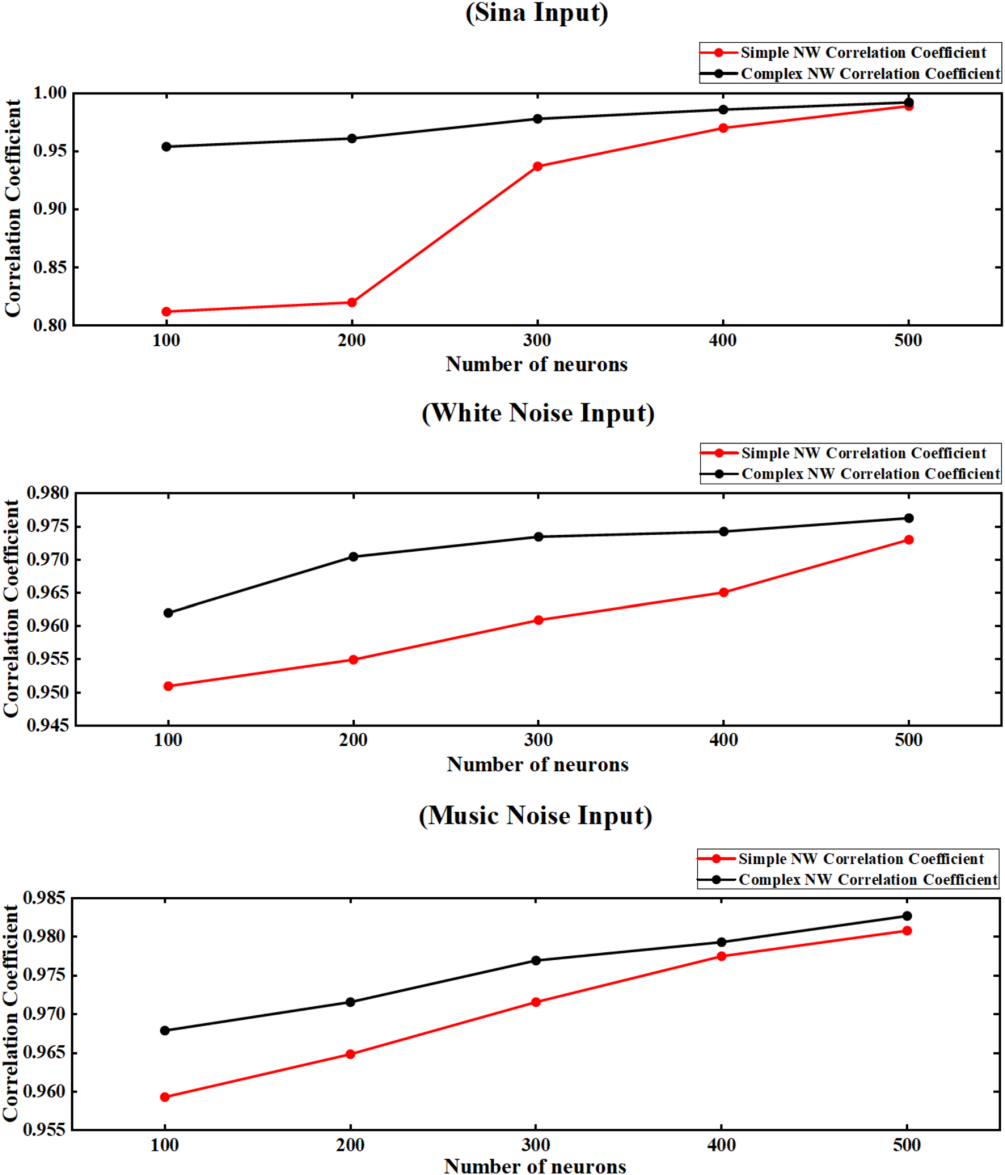
Comparison of correlation coefficients of neural networks with different input signals, different scales and different topologies.

In order to better compare the complexity of the network and the influence of the size of the neurons on the anti-interference characteristics of the network, the correlation coefficients of the simple NW neural network composed of 100, 200, 300, 400, 500 neurons with sinusoidal input, Gaussian white noise and music noise input and the complex NW neural network are drawn and listed, as shown in Fig. 28.

By comparing the Pearson correlation coefficients of simple NW small-world networks and complex NW small-world networks across scales (100-500 neurons) under sinusoidal signal, Gaussian white noise input and musical noise input, we reveal that increasing network size and topological connection complexity will enhance anti-jamming performance. The experimental data show that under sinusoidal input, the simple NW network increases from 0.812 of 100 neurons to 0.989 of 500 neurons (an increase of 21.8 %), and the complex NW network increases from 0.954 to 0.992 (an increase of 4.0 %). Under the input of Gaussian white noise, the simple NW network increased from 0.951 to 0.973 (an increase of 2.0 %), and the complex NW network increased from 0.962 to 0.976 (an increase of 1.5 %). Under the music noise input, the simple NW network increased from 0.959 to 0.981 (an increase of 2.0 %), and the complex NW network increased from 0.968 to 0.983 (an increase of 1.5 %). At the same time, complex NW networks always show better immunity. The complex topology optimizes the path characteristics between neurons through the connection mechanism, thereby enhancing the anti-interference of noise.

In summary, the topology and scale of the network play a decisive role in anti-disturbance performance. The simple ring network structure has weak immunity in the face of noise and large-scale network, but it still reflects the law that the correlation coefficient increases with the increase of scale. The NW small-world network can maintain high synchronization and anti-noise ability in large-scale networks through its good connection characteristics, showing excellent immunity.

## Discussion

In recent years, the research on the anti-interference characteristics of neuronal networks has gradually shifted from single neuron dynamics analysis to the exploration of network topology and synaptic coordination mechanism. Previous studies have made important progress in this field: early work revealed the relationship between chemical synapses and topological structure on disturbance rejection characteristics of neural network by constructing small-scale networks (such as 10-100 neurons). For example, research on simple ring networks shows that unidirectional chemical synaptic connections can achieve local signal synchronization under noise interference, but limited by network size and topology, signal transmission efficiency is weak. Further research compares the performance differences of different synaptic types (such as chemical synapses and electrical synapses) in the network, and finds that chemical synapses can better suppress noise interference in a specific topology, but the conclusion is still limited to small-scale networks, and the relationship between topology complexity and network scale expansion has not been studied in depth.

This study has made two advances on the basis of the existing work. Firstly, different from the previous studies on multi-synaptic types, this paper focuses on a single Leonid chemical synaptic model to construct a network framework, superimposes high-randomness music signals on the network, and gradually expands the network size from 100 to 500 neurons. It is found that the anti-interference performance of the neuronal network increases with the increase of the size of the neuronal network. Subsequently, the complexity of the network is gradually increased, and the simulation model of the complex NW small-world network is constructed for the first time. The research results show that the anti-interference ability of the neural network has a significant positive correlation with its structural complexity. The correlation coefficient of the complex NW small-world network is nearly four times higher than that of the ring network.

The limitation of this study is that the reliability of the simulation results has not been verified by hardware experiments. Although numerical simulation reveals the synergistic effect of scale expansion and topological complexity on anti-jamming performance, its realizability in physical systems still needs to be further explored. For example, redundant connections in complex NW small-world networks may introduce additional signal interference in hardware circuits. In the follow-up work, the neuromorphic hardware system is constructed based on the FPGA (Field Programmable Gate Array) platform, and the topological connection characteristics of the neural network are reproduced by parallel logic units. Such hardware reproduction experiments will provide practical theoretical support for the research of disturbance rejection characteristics of neural network.

## Conclusions

In this study, the anti-interference characteristics of three topologies (simple ring network, simple NW small world network and complex NW small world network) under different neuron sizes (100 ~ 500) are quantitatively analyzed by simulation modeling. The simulation results show that under the condition of noise-free sinusoidal excitation, with the increase of the number of neurons, the signal correlation index of the simple ring network increases significantly, from 0.121 in 100 neurons to 0.190 in 500 neurons, while the signal correlation index of the simple NW and complex NW small-world networks increases from 0.812 to 0.989 and 0.954 to 0.992, respectively. Under the Gaussian white noise interference environment, the correlation index of the simple ring network is increased from the initial value of 0.216 (100 neurons) to 0.223 (500 neurons), the simple NW small world network is optimized from 0.951 to 0.973, and the complex NW small world network is increased from 0.962 to 0.976. In the music noise interference environment, the correlation index of the simple ring network is increased from the initial value of 0.240 (100 neurons) to 0.661 (500 neurons), and the simple NW small world network is optimized from 0.959 to 0.981. The complex NW small-world network achieves the optimal performance of 0.983 at the maximum scale. The above data confirm that in the neural network constructed by Leonid chemical synapse and HH model, the expansion of neural network scale and the increase of topological connection complexity can effectively enhance the anti-interference ability of the system, and the complex NW small world network shows the highest anti-interference characteristics in both input modes.

Based on the current simulation results, the follow-up work intends to reproduce the network model through the hardware platform to verify the anti-interference characteristics of the neural network in the hardware system, and provide experimental support for the reliability of the brain-like simulation model. At the same time, we will explore more regulation rules of topological structure on correlation coefficient, and improve the research of artificial neural network simulation. We will further systematically study the influence of different signal amplitude and noise intensity on the anti-interference performance of neural networks of different scales, so as to enrich and improve the research conclusions of this problem.

## Data Availability

All data are available from the corresponding author upon request.
